# Complication drivers in head and neck free flap surgery: A single-center predictive analysis

**DOI:** 10.1007/s10006-026-01512-0

**Published:** 2026-03-21

**Authors:** Jakob Fenske, Leonard Knoedler, Michael Alfertshofer, Friedrich Mrosk, Philipp Lampert, Christian Doll, Kilian Kreutzer, Max Heiland, Steffen Koerdt, Carsten Rendenbach

**Affiliations:** Department of Oral and Maxillofacial Surgery, Charité – Universitätsmedizin Berlin, Corporate Member of Freie Universität Berlin, Humboldt-Universität zu Berlin, Berlin, Germany

**Keywords:** Free flap reconstruction, Outcome analysis, Microvascular reconstruction, Maxillofacial surgery, Prior radiotherapy, Surgeon experience

## Abstract

**Purpose:**

Head and neck free flap (HNFF) surgery is essential in reconstructive surgery, addressing complex defects with tailored approaches. Despite high success rates, complications remain significant, influenced by patient comorbidities, flap types, and surgical complexity. This study aimed to analyze predictors of postoperative complications to improve patient outcomes.

**Methods:**

This monocentric retrospective cohort study included patients undergoing microvascular free flap reconstruction for head and neck defects between April 2017 and July 2023. The primary outcome was flap-specific postoperative complications, including flap loss, recipient site wound healing disorders (WHD), partial flap necrosis, or anastomotic insufficiency.

**Results:**

Among 1050 patients, the free flap success rate was 94%, with 59 flap losses. Complications occurred in 44% of patients, most commonly WHD (29%). Prior head and neck radiotherapy was associated with anastomosis revision (OR 2.13; *p* = 0.02) and flap loss (OR 2.06; *p* = 0.02). Female sex increased the risk of flap loss (OR 2.24; *p* = 0.004). Alcohol abuse elevated the risk of WHD (OR 1.81; *p* = 0.003) and partial flap necrosis (OR 2.19; *p* = 0.001). Infection risk was influenced by age (OR 0.98; *p* = 0.01), bleeding disorders (OR 5.37; *p* = 0.003), and prior microvascular reconstruction (OR 2.08; *p* = 0.008). Increased surgical experience only slightly correlated with higher flap loss rates (OR 1.06; *p* = 0.02).

**Conclusion:**

Free flap surgery is effective in head and neck reconstruction, though specific risk factors contribute to complications. Further research is needed to mitigate these risks. Surgical experience is essential for complex cases but is not a primary, clinically relevant predictor of complications in high-volume training centers.

## Introduction

 Facial reconstructive surgery represents a growing surgical branch and includes an array of surgical procedures, from skin autografts to facial allotransplantation [1–3]. Free flap surgery is considered a workhorse in reconstructive surgery. Over the past decades, the clinical need for head and neck free flap surgery has been steadily increasing, catalyzed by the rising incidence of head and neck malignancies and facial trauma [4, 5]. Head and neck free flaps (HNFFs) provide a versatile platform to restore form and functionality in patients with complex craniofacial defects. Different types of HNFFs are commonly used in head and neck reconstruction, offering a tailored treatment based on the anatomical and functional profile of the defect [6].

Among the most commonly used transplants, osseous flaps involve fibula free flap (FFF), scapula free flap (SFF) and the deep circumflex iliac artery free flap (DCIA). Regularly employed non-osseous flaps involve the radial free flap (RFF), the anterolateral thigh flap (ALT) and the latissimus free flap (LFF). Of note, there are many more options to provide tailored reconstructive surgery.

Head and neck free flap surgery is generally considered safe and shows high success rates. However, depending on patient comorbidities, flap type, and surgical complexity, reported complication rates and outcomes can vary significantly, with complication rates as high as 85% [7, 8]. Common complications include complete/partial flap loss, infection, and wound dehiscence, which can significantly impact patient outcomes [9]. To reduce complications, different perioperative concepts have been proposed, including better postoperative monitoring strategies, improved intraoperative care, as well as prehabilitation [10–12]. Preoperative patient screening represents a potent strategy to determine the patient’s eligibility for surgery and identify patients at risk of perioperative complications. Retrospective data analysis can help unravel novel risk factors, refine preoperative patient screening and tailor perioperative care. Numerous recent studies provided evidence for various predictive factors of complications, such as diabetes mellitus, surgery duration and nutritional parameters, as well as history of head and neck surgery, recipient vessels and tools in the course of microvascular anastomoses and patient age [13–16].

However, there is a paucity of research that leverages high numbers of HNFF procedures to analyze outcomes and deduce risk factors. This gap leaves untapped potential to improve outcomes following HNFFs. Therefore, the aim of this study was to analyze outcomes and predictors for flap complications in a high-volume university medical center.

## Materials and methods

### Patient selection

Ethical approval was obtained by the local ethics committee (EA2/138/18). The study was performed in accordance with the Declaration of Helsinki. In this monocentric, retrospective cohort study, all patients who underwent reconstructive microvascular free flap surgery for reconstruction of head and neck defects at the Department of Oral and Maxillofacial Surgery at the Charité Universitätsmedizin Berlin, Germany between April 2017 and July 2023, were deemed eligible. The inclusion criteria were: (1) patients receiving microvascular free flaps for (2) head and neck reconstruction, who were (3) at least 18 years of age at the time of surgery. Patients with (1) missing therapy documentation, (2) non-titanium osteosynthesis plates and (3) two or more synchronous flaps were excluded from the study cohort.

### Analyzed variables

The primary endpoint was the occurrence of postoperative recipient site complications in the area of the transplanted microvascular free flap (anastomosis revision, flap loss, recipient site wound healing disorders (WHD), partial necrosis, infection and bone exposure in osseous flaps). WHD were defined as any postoperative impairment of physiological wound healing at the flap recipient site documented in the medical records. This included wound dehiscence, delayed wound healing requiring prolonged local treatment, secondary wound closure or surgical revision, as well as clinically relevant wound breakdown without signs of acute infection. WHD were recorded based on routine clinical documentation and were analyzed as a composite endpoint reflecting real-world postoperative wound healing complications. Patient (sex, age, constitutional data, comorbidities, medication, surgical indication) and treatment characteristic (flap type and surgery duration) as well as follow-up documentations were collected until July 2024. The surgeons’ experience was evaluated by documenting the years of experience following board certification.

### Statistical analysis

Statistical calculations were performed using SPSS, Version 29 (IBM Corp.). Results were considered statistically significant if the p-value was < 0.05. Inclusion of the null value in the 95%-confidence interval (CI) of odds ratios (OR) was recorded as non-significant, while non-inclusion was recorded as significant. All variables were analyzed in univariate analysis for flap complications as dependent categorical variables. The dependent variable “any complication” was defined as the occurrence of at least one of the other complications in a patient. Qualitative variables were compared using the Chi-squared test or Fisher’s exact test, if the expected cell counts were below five. Quantitative variables were first assessed for normality using the Shapiro-Wilk test and subsequently compared using Mann-Whitney U test to compare medians between the groups. Parameters that showed significant differences were subsequently included in multivariate models to adjust for confounders. Specifically, binary logistic regressions were used. The predictive power of these models was assessed by calculating the area under the curve (AUC) of the receiver operating characteristic (ROC) curve. The same approach was then taken to perform subgroup analyses for osseous and non-osseous flaps, and malignant and non-malignant surgery indications, respectively.

## Results

### Preoperative patient demographics and health characteristics

This study included 1,050 patients (429 females, 621 males) with a mean age of 65.6 ± 13.5 years and a mean BMI of 24.6 ± 4.8 kg/m². The average duration of hospitalization was 17.8 ± 11.9 days. The most prevalent comorbidities were hypertension (44.5%), nicotine abuse (33.2%), and alcohol abuse (17.4%). In terms of medication use, antihypertensives were prescribed in 43.6% of patients, followed by statins in 22.3%, and anticoagulants in 18.3%.

### Surgical characteristics

The overall average surgical duration was 505.0 ± 148.1 min. Among malignant indications, primary reconstructions had an average of 508.2 ± 145.1 min, while secondary reconstructions took 450.5 ± 185.6 min. Surgeries for non-malignant indications lasted 512.3 ± 140.4 min. Flapwise, surgeries including osseous flaps lasted 579.7 ± 145.1 min, while those involving non-osseous flaps took 456.6 ± 129.5 min. The primary indication for free flap surgery was the treatment of malignant tumors, which accounted for 83.8% of cases. A history of radiotherapy was reported in 18.1% of patients, while 11.4% had undergone prior chemotherapy. RFF were the most commonly used flap (45.5%), followed by FFF (33.0%) and ALT (9.9%). The mean surgical experience of the main responsible surgeon was 6.5 ± 5.2 years (Table 1).

**Table 1:** Patient characteristics. (*BMI = body mass index*,* ORN = osteoradionecrosis*,* MRONJ = medication associated osteonecrosis of the jaw*,* FFF = fibula free flap*,* SFF = scapula free flap*,* DCIA = deep circumflex Iliac artery free flap*,* RFF = radial free flap*,* ALT = anterolateral thigh flap*,* LFF = latissimus free flap*,* INR = International normalized Ratio*,* PTT = partial thromboplastin time*,* GFR = glomerular filtration rate*)

	Overall		Overall
	*N* = 1050 (100.0%)		*N* = 1050 (100.0%)
Age (mean in years)	65.6 ± 13.5	**Surgical experience (mean in years)**	6.5 ± 5.2
Female	429 (40.9%)	**Hospitalization (days)**	17.8 ± 11.9
Male	621 (59.1%)	**Flap type**	
BMI (kg/m ^2^)	24.6 ± 4.8	FFF	346 (33.0%)
Comorbidities		SFF	45 (4.3%)
Nicotine abuse	349 (33.2%)	DCIA	18 (1.7%)
Alcohol abuse	183 (17.4%)	RFF	478 (45.5%)
Hypertension	467 (44.5%)	ALT	104 (9.9%)
Diabetes mellitus	140 (13.3%)	LFF	41 (3.9%)
Arteriosclerosis	154 (14.7%)	Parascapular Flap	5 (0.5%)
History of thrombosis	38 (3.6%)	Other	13 (1.2%)
Bleeding disorder	14 (1.3%)	**Surgery duration (minutes)**	505.0 ± 148.1
Hypothyroidism	162 (15.4%)	**Preoperative blood parameters**	
Indication		Hemoglobin [g/dl]	13.1 ± 2.0
Malignant tumor	880 (83.8%)	INR	1.2 ± 0.3
ORN	75 (7.1%)	PTT [s]	31.2 ± 7.2
Benign tumor	22 (2.1%)	Thrombocytes [N/µl]	268,198.2 ± 89,993.7
Osteomyelitis	19 (1.8%)	GFR [ml/min]	78.9 ± 17.7
MRONJ	18 (1.7%)	**Medication**	
Trauma	9 (0.9%)	Anticoagulants	193 (18.3%)
Other	27 (2.6%)	Antiplatelet drug	7 (0.7%)
Prior Head and Neck Radiotherapy	190 (18.1%)	Statins	234 (22.3%)
Prior Systemic Chemotherapy	120 (11.4%)	Antihypertensives	458 (43.6%)
Prior Microvascular Free Flap	180 (17.1%)	**Follow-up (months)**	25.5 ± 20.3

### Peri- and postoperative outcomes

In total, 460 (43.8%) patients experienced at least one complication with the most common being recipient site WHD in 307 patients (29.2%). Total flap loss occurred in 59 patients (5.6%). The overall free flap success rate was 94.4%, 89.3% in osseous flaps, 97.3% in non-osseous flaps, 94.8% in malignant and 92.4% in non-malignant indications.

In the univariate analysis, age demonstrated a statistically significant association with the occurrence of wound infections (*p* < 0.001). Nicotine abuse and alcohol abuse were identified as significant risk factors for flap loss (*p* = 0.02 and *p* = 0.006, respectively) and WHD (*p* = 0.002 and *p* < 0.001, respectively). Wound infections were most frequently associated with malignant tumors (*p* = 0.03) and ORN (*p* = 0.01) as surgical indications. A history of prior head and neck radiotherapy markedly increased the risk of anastomosis revision (*p* = 0.003), flap loss (*p* = 0.03), and infection (*p* < 0.001). Notably, patients with a history of prior microvascular flap reconstruction exhibited a further increased risk of infection (*p* < 0.001).

**Fig. 1:**
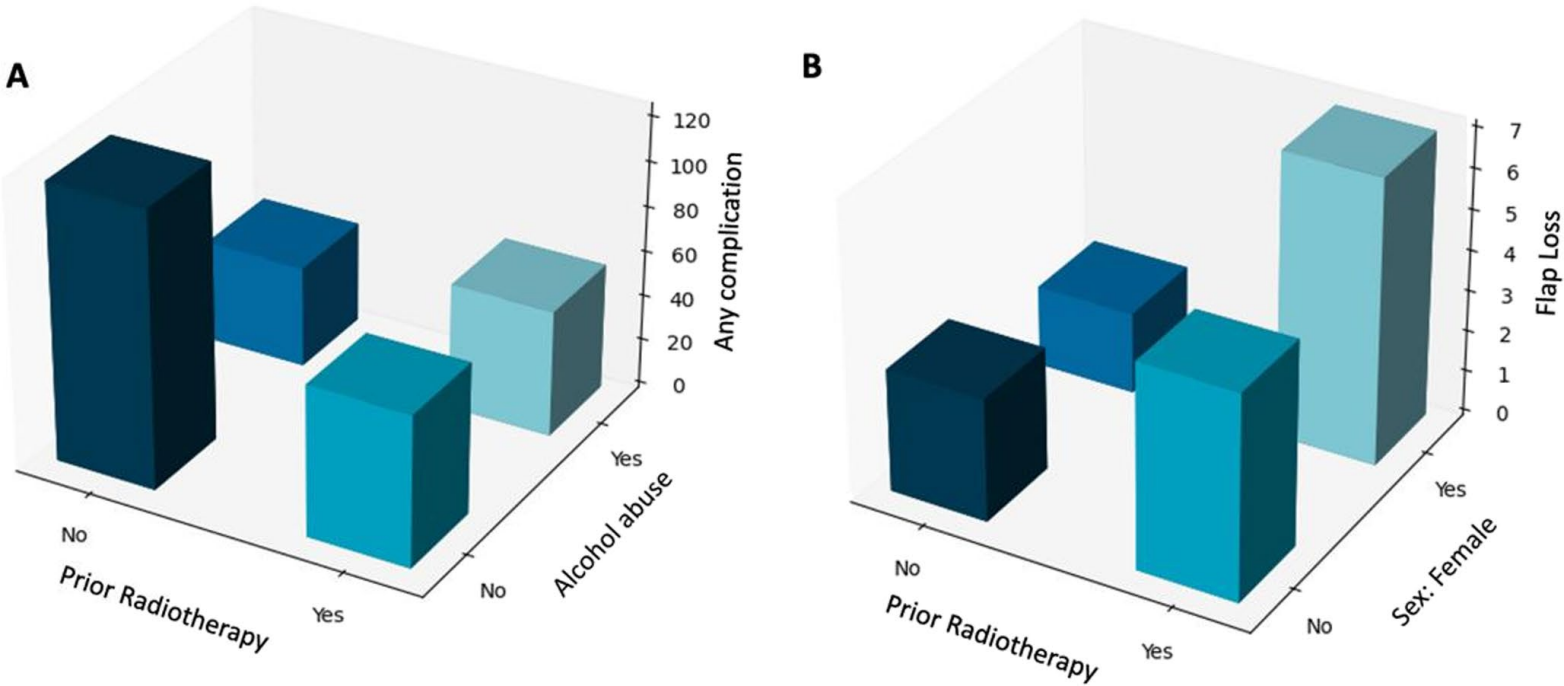
3D-Bar frequency plots with main predictors for (**A**) any complication and (**B**) flap loss

In the multivariate analysis, a history of prior head and neck radiotherapy remained a strong independent risk factor for anastomosis revision (OR 2.13; 95% CI [1.13;4.01]; *p* = 0.02) and flap loss (OR 2.06; 95% CI [1.13;3.73]; *p* = 0.02). Alcohol abuse increased the risk of WHD (OR 1.81; 95% CI [1.22;2.69]; *p* = 0.003) and partial flap necrosis (OR 2.19; 95% CI [1.36;3.52]; *p* = 0.001). Increased surgical experience was statistically slightly associated with flap loss (OR 1.06; 95% CI [1.01;1.11]; *p* = 0.02) and osseous flap loss (OR 1.09; 95% CI [1.02;1.16]; *p* = 0.007) (Tables 2 and 3, Appendix Table A5, Fig. 1).

**Table 2:** Univariate analysis of preoperative parameters on dependent variables. (*WHD = wound healing disorder*,* BMI = body mass index*,* ORN = osteoradionecrosis*,* MRONJ = medication associated osteonecrosis of the jaw*,* INR = International normalized Ratio*,* PTT = partial thromboplastin time*,* GFR = glomerular filtration rate*)

	Any Complication (*n* = 460) Yes/No (*p*)	Anastomosis Revision (*n* = 54) Yes/No (*p*)	Flap Loss (*n* = 59) Yes/No (*p*)	WHD (*n* = 307) Yes/No (*p*)	Partial Necrosis (*n* = 94) Yes/No (*p*)	Infection (*n* = 148) Yes/No (*p*)
Age (mean in years)	65.3/65.9 (0.21)	63.6/65.7 (0.67)	63.1/65.8 (0.08)	65.2/65.8 (0.18)	64.1/65.7 (0.14)	63.1/66 (***< 0.001***)
Female	173/287 (0.06)	29/25 (0.05)	32/27 (***0.03***)	116/191 (0.19)	31/63 (0.10)	51/97 (0.09)
Male	287/173 (0.06)	25/29 (0.05)	27/32 (***0.03***)	191/116 (0.19(	63/31 (0.10)	97/51 (0.09)
BMI (kg/m ^2^)	24.0/24.8 (***0.04***)	23.4/24.5 (0.35)	24.4/24.5 (0.62)	24.0/24.7 (0.07)	23.4/24.6 (0.05)	23.8/24.6 (0.16)
Comorbidities						
Nicotine abuse	171/289 (***0.02***)	12/42 (0.08)	28/31 (***0.02***)	124/183 (***0.002***)	34/60 (0.53)	57/91 (0.14)
Alcohol abuse	104/356 (***< 0.001***)	3/51 (***0.02***)	18/41 (***0.006***)	78/229 (***< 0.001***)	28/66 (***< 0.001***)	34/113 (0.06)
Hypertension	204/256 (0.94)	23/31 (0.78)	22/37 (0.25)	139/168 (0.74)	41/53 (0.86)	64/84 (0.75)
Diabetes mellitus	62/398 (0.90)	7/47 (0.93)	10/49 (0.40)	47/260 (0.23)	13/81 (0.88)	21/127 (0.74)
Arteriosclerosis	83/377 (***0.006***)	10/44 (0.41)	12/47 (0.21)	57/250 (***0.02***)	20/74 (0.06)	25/123 (0.41)
History of thrombosis	17/443 (0.91)	1/53 (0.71)	4/55 (0.18)	12/295 (0.75)	3/91 (1.0)	10/138 (***0.03***)
Bleeding disorder	8/452 (0.31)	1/53 (0.53)	1/58 (0.56)	3/304 (0.77)	0/94 (0.63)	6/142 (***0.002***)
Hypothyroidism	68/392 (0.61)	12/42 (0.16)	13/46 (0.15)	47/260 (0.95)	13/81 (0.65)	30/118 (0.08)
Indication						
Malignant tumor	379/81 (0.27)	43/11 (0.39)	46/13 (0.21)	261/46 (0.50)	75/19 (0.27)	115/33 (***0.03***)
ORN	39/421/0.14)	6/48 (0.27)	8/51 (0.05)	22/285 (0.99)	9/85 (0.34)	18/130 (***0.01***)
Benign tumor	9/451 (0.78)	1/53 (1.0)	2/57 (0.48)	7/300 (0.79)	2/92 (1.0)	0/148 (0.06)
Osteomyelitis	13/447 (***0.03***)	2/52 (0.26)	2/57 (0.35)	8/299 (0.21)	1/93 (1.0)	4/144 (0.33)
MRONJ	5/455 (0.17)	1/53 (0.62)	0/59 (0.62)	4/303 (0.51)	2/92 (0.67)	4/144 (0.30)
Trauma	4/456 (0.97)	0/54 (1.0)	0/59 (1.0)	2/305 (0.64)	1/93 (0.57)	3/145 (0.12)
Other	11/449 (0.75)	1/53 (1.0)	1/58 (0.66)	3/304 (***0.04***)	4/90 (0.29)	4/144 (0.79)
Preoperative blood parameters						
Hemoglobin [g/dl]	13.0/13.1 (0.35)	12.5/13.2 (***0.02***)	13.0/13.1 (0.39)	13.0/13.1 (0.99)	13.2/13.1 (0.71)	13.1/13.1 (0.36)
INR	1.2/1.2 (0.64)	1.2/1.2 (0.87)	1.2/1.2 (0.61)	1.2/1.2 (0.51)	1.2/1.2 (0.86)	1.2/1.2 (0.91)
PTT [s]	31.1/31.3 (0.32)	31.4/31.2 (0.25)	30.4/31.3 (0.94)	31.1/31.3 (0.63)	31.5/31.2 (0.49)	31.6/31.1 (0.10)
Thrombocytes [N/µl]	265,480/270,464 (0.72)	252,680/268,853 (0.40)	266,125/268,211 (0.87)	274,190/265,624 (0.15)	262,827/268,595(0.32)	264,539/268,677 (0.84)
GFR [ml/min]	78.8/79.1 (0.95)	83.3/78.6 (0.29)	80.7/78.7 (0.38)	78.9/78.8 (0.52)	80.3/78.7 (0.28)	80.8/78.5 (0.06)
Medication						
Statins	107/353 (0.50)	14/40 (0.51)	12/47 (0.71)	81/226 (***0.04***)	15/79 (0.12)	36/112 (0.52)
Antihypertensives	193/267 (0.34)	20/34 (0.32)	19/40 (0.07)	136/171 (0.78)	38/56 (0.51)	54/94 (0.06)
Anticoagulants	50/410 (0.87)	10/33 (0.07)	7/52 (0.84)	28/279 (0.20)	8/86 (0.41)	12/136 (0.22)
Antiplatelet drug	86/374 (0.82)	10/44 (0.98)	11/48 (0.96)	57/250 (0.92)	18/76 (0.84)	24/124 (0.46)
Prior Head and Neck Radiotherapy	96/364 (***0.04***)	18/36 (***0.003***)	17/42 (***0.03***)	57/250 (0.80)	23/71 (0.09)	42/106 (***< 0.001***)
Prior Systemic Chemotherapy	60/400 (0.15)	9/45 (0.21)	10/49 (0.17)	38/269 (0.53)	15/79 (0.15)	26/122 (***0.01***)
Prior Microvascular Free Flap	78/382 (0.50)	14/40 (***0.04***)	11/48 (0.58)	53/254 (0.51)	14/80 (0.74)	40/108 (***< 0.001***)
Surgical experience (mean in years)	6.7/6.3 (0.38)	7.0/6.4 (0.44)	7.9/6.4 (***0.004***)	6.0/6.7 (0.15)	6.9/6.4 (0.10)	7.2/6.3 (***0.01***)

**Table 3:** Multivariate analysis of preoperative parameters on dependent variables. (*WHD = wound healing disorder*, *ORN = osteoradionecrosis*,* OR = Odds Ratio*,* CI = confidence interval*,* ROC-AUC = receiver operating characteristics – area under the curve*)

	OR [95%-CI]	*p*	ROC-AUC [95%-CI]
Any complication			0.58 [0.55;0.62]
BMI	0.98 [0.95;1.00]	0.09	
Nicotine abuse	1.04 [0.77;1.42]	0.79	
Alcohol abuse	1.84 [1.26;2.68]	***0.002***	
Indication: Osteomyelitis	3.15 [1.18;8.42]	***0.02***	
Prior Head and Neck Radiotherapy	1.39 [1.01;1.92]	***0.04***	
Anastomosis Revision			0.67 [0.59;0.75]
Alcohol abuse	0.30 [0.09;0.98]	0.05	
Hemoglobin	0.87 [0.76;1.01]	0.06	
Prior Head and Neck Radiotherapy	2.04 [1.03;4.06]	***0.04***	
Prior Microvascular Free Flap	1.13 [0.53;2.39]	0.75	
Flap Loss			0.71 [0.65;0.76]
Female	2.27 [1.31;3.94]	***0.004***	
Nicotine abuse	1.70 [0.88;3.30]	0.12	
Alcohol abuse	2.06 [1.00;4.26]	0.05	
Prior Head and Neck Radiotherapy	2.06 [1.13;3.73]	***0.03***	
Surgical experience	1.06 [1.01;1.11]	***0.02***	
WHD			0.61 [0.57;0.64]
Nicotine abuse	1.19 [0.85;1.66]	0.31	
Alcohol abuse	1.81 [1.22;2.69]	***0.003***	
Arteriosclerosis	1.33 [0.90;1.96]	0.16	
Indication: Other	0.31 [0.09;1.03]	0.06	
Statins	1.27 [0.91;1.78]	0.17	
Partial Necrosis			0.57 [0.51;0.63]
Alcohol abuse	2.19 [1.36;3.52]	***0.001***	
Infection			0.65 [0.60;0.70]
Age	0.98 [0.97;0.99]	***0.01***	
History of Thrombosis	2.21 [1.03;4.75]	***0.04***	
Bleeding disorder	4.95 [1.63;15.06]	***0.005***	
Malignant tumor	1.06 [0.57;1.97]	0.86	
ORN	1.30 [0.53;3.22]	0.57	
Prior Head and Neck Radiotherapy	1.46 [0.79;2.71]	0.23	
Prior Systemic Chemotherapy	0.98 [0.52;1.87]	0.96	
Prior Microvascular Flap	1.68 [1.04;2.69]	***0.03***	
Surgical experience	1.02 [0.99;1.05]	0.27

Appendix Tables A1, A2, A3, A4, A5, A6, A7, A8 summarize the uni- and multivariate subgroup analyses for osseous and non-osseous flaps as well as for malignant and non-malignant indications.

## Discussion

Free flap surgery represents a cornerstone of reconstructive surgery. HNFFs provide a valuable reconstructive option for a broad range of clinical indications (e.g., oncological surgery, trauma). Here, we analyzed 1,050 HNFF cases to investigate outcomes and risk factors of HNFF surgery.

In this study, the overall free flap success rate was 94%, which is consistent with previous research work. For instance, a 2006 US study enrolled 735 free tissue transfers, including, among others, head and neck flaps. The authors stated a free flap success rate over of 90% [17]. Kwok et al. accessed a multi-center database and identified 1,187 microvascular free tissue transfers. While the overall flap failure rate was 5.1%, HNFFs had the highest rate of free flap failure at 7.7% [18]. Eckart and Fokas reviewed 500 HNFF cases over an 18-year period and found that the total flap loss rate was 6%, converting to a free flap success rate of 94% [19]. In two national single-center studies, flap loss rates of 6.2% and 4.9% were reported [20, 21]. On a larger scale, Katsnelson et al. used a multi-centric and multi-national database and reported free flap success rates of more than 90% when reviewing 1,297 HNFF patients [22]. While it is important to note that multiple parameters (e.g., patient cohort, perioperative monitoring, follow-up, donor/recipient region) impact flap failure/success, our free flap success rates align with previous research in the field. Collectively, this finding underscores the clinical safety of HNFFs while uncovering untapped potential to further improve postoperative outcomes.

Diving deeper into postoperative outcomes, we found an overall complication rate of 44% with wound healing disorders being the most common adverse events. In contrast to flap success rates, complication rates of HNFFs show a wide heterogeneity, strongly depending on the clinical indication and patient population. In general, complication rates following such procedures oscillate between 40 and 50% [23–25]. For example, Lee et al. systematically reviewed the existing literature on microvascular free flap reconstruction for mandibular osteoradionecrosis. The authors reported postoperative complications in 39.7% of 368 cases [26]. Further, a review of complications in head and neck free flap reconstruction by Eskander et al. found a total complication rate of 54% [16]. On the lower end, a recent meta-analysis by McGregor et al. included 612 scapula free flaps with a pooled postoperative complication rate of 10.7% [27]. Similarly, Zhang et al. found a complication rate of 16.5% in 110 patients who underwent ALT flap surgery [28]. Further, Kroll et al. reported adverse events in 4 of 30 oncological head and neck patients who received a rectus abdominis free flap, resulting in a complication rate of 13% [29]. However, there are also studies with complication rates higher than 60% and even up to 85% [24, 30]. Overall, our study highlights the persisting need to further improve perioperative patient management in HNFF collectives and reduce perioperative complications following HNFF surgery.

Identifying preoperative risk factors and revising current patient screening algorithms can support preoperative decision-making and help reduce perioperative risk factors. Our study highlights, that numerous preoperative parameters are capable of affecting the outcome of free flap surgery. However, adjusted models and subgroup analyses revealed alcohol abuse, prior head and neck radiotherapy and previously received microvascular free flaps as independent risk factors across multiple categories. Specifically, the well-established malignant risk factor alcohol abuse emerged as an even stronger predictor for complications such as WHD or partial necrosis compared to nicotine abuse. While alcohol is known to exhibit a variety of toxic effects on tissues, it has been previously linked to free flap loss due to its capability for vascular damage [15]. This pathophysiological concept might also apply to increased rates of WHD and partial flap necrosis, as they have been linked to alcohol abuse as well [14]. In this regard, the recently introduced concept of prehabilitation in various surgical disciplines may provide a perspective to further reduce alcohol-associated surgical risks [31]. Moreover, a history of head and neck radiotherapy and prior received microvascular flaps emerged as predictors for anastomosis revision, flap loss and wound infection, respectively. Radiotherapy is known to cause vascular pathologies and tissue fibrosis that compromise healing and complicate surgical procedures, but has not been directly linked to flap loss before [15, 32]. Similarly, the presence of scar tissue and previously anastomosed head and neck vessels in patients with prior microvascular free flaps poses technical challenges for surgeons and wound healing, possibly making these flaps more prone to complications. Notably, Zhou et al. associated free flap surgery in previously operated recipient sites with an increased risk of free flap failure [13]. These findings suggest that prior radiotherapy and previous free flap surgeries may be regarded as surrogate parameters for flap complications, indicating extensive tissue damage and prolonged therapies to be disadvantageous for free flap success. Although several identified risk factors such as alcohol abuse or prior radiotherapy are well established and not easily modifiable, the value of large cohort analyses lies in refining risk stratification rather than proposing novel treatment paradigms. surgeons. Additionally, the influence of surgical experience on free flap outcomes in a high-volume medical center was examined. This factor has rarely been analyzed in head and neck free flap surgery at this scale. While most parameters were not influenced by surgical experience, flap losses, especially in osseous flaps, statistically occurred slightly more often with increasing experience. However, this association requires cautious interpretation. Although statistical significance was reached, the effect size was minimal and unlikely to be clinically relevant. Given the retrospective design of the study, this finding should not be interpreted as evidence of a causal relationship. Rather, surgeon experience appears to play a subordinate role compared with patient- and procedure-related factors in a high-volume training center. While this correlation could be attributable to the increasing complexity of cases, e.g. extensive osseous reconstructions in pre-operated and pre-irradiated tissues, experienced surgeons are confronted with, it could also be a consequence of deteriorating meticulous attention or skills. Simultaneously, this observation also elucidates that a structured high-volume training center, emphasizing continuing education, allows for mostly experience-independent complication rates. These results are congruent with other studies, concluding that involving early-stage surgeons in microvascular surgery is a safe procedure [21, 33, 34]. Thus, the present data support the concept that optimized institutional structures may partially mitigate individual surgeon-related risk. Beyond that, these results are clinically relevant for patient counseling, benchmarking of outcomes, and the organization of training and supervision in high-volume centers. Furthermore, our results from subgroup analyses indicate that preoperative risk factors may be more robust for osseous free flap complications compared to non-osseous free flap complications, as many of the latter lacked predictors in univariate analysis. Our findings add to previously identified preoperative risk factors for free flap surgeries such as hypoalbuminemia and elevated frailty scores [35], diabetes and obesity [14, 36] or nicotine abuse and vascular diseases [37].

In summary, our results uncover a variety of notable risk factors for free flap surgery and underscore the importance of cohort- and procedure-adapted subgroup screening and subsequent thorough preoperative risk evaluation.

## Limitations

This study is not without limitations. The retrospective study design might have introduced unconsidered bias (e.g., recall bias) and confounding factors. Differences in accuracy and uniformity of data collection within and between the participating surgeons may represent additional limitations. Moreover, the database does not capture randomized data in a closer sense of data collected from randomized controlled trials. Further, the single-center study design might have impacted outcome analysis and interpretation, while lacking external validation of the findings presented herein. While our cohort encompassed 1,050 HNFF patients, a larger patient population can help uncover additional risk factors and clinical insights.

## Conclusion

This single-center retrospective analysis leveraged a database of 1,050 free flap surgeries for reconstruction of maxillofacial defects. While 44% of patients experienced any type of flap complication, the overall flap success rate was 94%. Alcohol abuse, history of head and neck radiotherapy and previously received free flaps were identified as potent preoperative risk factors across a variety of flap complications. Surgical experience is essential for managing complex reconstructions but was not identified as a clinically relevant independent predictor of flap loss. Profound patient screening is advised to further optimize risk-adapted head and neck free flap surgery.

## Data Availability

The data that support the findings of this study are not openly available due to reasons of sensitivity and are available from the corresponding author upon reasonable request.

## Appendix

**Table 4:** Univariate subgroup analysis for osseous flaps of preoperative parameters on dependent variables. (*WHD = wound healing disorder*,* BMI = body mass index*,* ORN = osteoradionecrosis*,* MRONJ = medication associated osteonecrosis of the jaw*,* INR = International normalized Ratio*,* PTT = partial thromboplastin time*,* GFR = glomerular filtration rate*)

	Any Complication (*n* = 210) Yes/No (*p*)	Anastomosis Revision (*n* = 27) Yes/No (*p*)	Flap Loss (*n* = 42) Yes/No (*p*)	WHD (*n* = 133) Yes/No (*p*)	Partial Necrosis (*n* = 46) Yes/No (*p*)	Infection (*n* = 106) Yes/No (*p*)	Bone exposure (*n* = 74) Yes/No (*p*)
Age (mean in years)	62.4/62.2 (0.15)	60.2/62.5 (0.64)	62.3/62.3 (0.58)	62.9/62.0 (0.61)	60.5/62.5 (0.14)	62.8/62.1 (0.54)	60.7/62.7 (***0.009***)
Female	81/129 (0.66)	18/9 (***0.003***)	21/21 (0.15)	51/82 (0.72)	20/26 (0.57)	34/72 (0.07)	24/50 (0.16)
Male	129/81 (0.66)	9/18 (***0.003***)	21/21 (0.15)	82/51 (0.72)	26/20 (0.57)	72/34 (0.07)	50/24 (0.16)
BMI (kg/m ^2^)	23.2/24.5 (***0.04***)	21.7/24.0 (0.10)	23.4/23.9 (0.64)	23.1/24.2 (0.09)	22.5/24.0 (0.08)	23.4/24.0 (0.34)	22.9/24.1 (0.05)
Comorbidities							
Nicotine abuse	93/117 (***0.02***)	6/21 (0.07)	21/21 (0.12)	66/67 (***0.002***)	16/30 (0.55)	47/59 (0.18)	44/30 (***< 0.001***)
Alcohol abuse	54/156 (***< 0.001***)	2/25 (0.17)	13/29 (***0.03***)	42/91 (***< 0.001***)	13/33 (0.08)	27/79 (***0.04***)	30/44 (***< 0.001***)
Hypertension	84/126 (0.87)	12/15 (0.60)	16/26 (0.83)	58/75 (0.25)	16/30 (0.48)	45/61 (0.49)	25/49 (0.26)
Diabetes mellitus	27/183 (0.16)	5/22 (0.19)	9/33 (***0.02***)	17/116 (0.40)	3/43 (0.45)	16/90 (0.09)	9/65 (0.67)
Arteriosclerosis	34/176 (***0.03***)	5/22 (0.37)	8/34 (0.19)	24/109 (***0.03***)	9/37 (0.14)	18/88 (0.13)	9/65 (0.88)
History of thrombosis	12/198 (0.29)	1/26 (0.81)	3/39 (0.43)	8/125 (0.36)	1/45 (0.71)	6/100 (0.59)	5/69 (0.36)
Bleeding disorder	8/202 (0.46)	1/26 (0.87)	1/41 (1.0)	3/130 (0.56)	0/46 (0.38)	6/100 (0.11)	3/71 (0.71)
Hypothyroidism	44/166 (***0.03***)	8/19 (0.11)	9/33 (0.43)	28/105 (0.14)	12/34 (0.09)	25/81 (***0.04***)	20/54 (***0.01***)
Indication							
Malignant tumor	152/58 (0.91)	18/9 (0.51)	31/11 (0.80)	100/33 (0.34)	32/14 (0.68)	82/24 (0.16)	54/20 (0.86)
ORN	31/179 (0.62)	5/22 (0.56)	7/35 (0.59)	17/116 (0.64)	7/39 (0.79)	16/90 (0.69)	8/66 (0.39)
Benign tumor	8/202 (0.41)	1/26 (1.0)	2/40 (1.0)	5/128 (0.55)	2/44 (1.0)	0/106 (***0.005***)	4/70 (0.76)
Osteomyelitis	10/200 (0.53)	2/25 (0.31)	2/40 (0.69)	7/126 (0.44)	1/45 (0.48)	3/103 (0.58)	3/71 (1.0)
MRONJ	5/205 (0.69)	1/26 (0.53)	0/42 (0.61)	3/130 (1.0)	2/44 (0.36)	3/103 (1.0)	4/70 (0.12)
Trauma	3/207 (0.95)	0/27 (1.0)	0/42 (1.0)	1/132 (0.67)	1/46 (0.51)	2/104 (0.65)	1/73 (1.0)
Other	1/209 (0.29)	0/27 (1.0)	0/42 (1.0)	0/133 (0.31)	1/45 (0.38)	0/106 (0.58)	0/74 (1.0)
Preoperative blood parameters							
Hemoglobin [g/dl]	13.1/12.8 (0.21)	12.5/13.0 (0.24)	13.3/12.9 (0.41)	13.1/13.0 (0.36)	13.2/12.9 (0.73)	13.3/12.8 (***0.02***)	13.5/12.9 (***0.04***)
INR	1.2/1.2 (0.58)	1.2/1.1 (1.0)	1.2/1.1 (0.38)	1.1/1.2 (0.77)	1.1/1.2 (0.83)	1.2/1.1 (0.88)	1.1/1.2 (0.08)
PTT [s]	30.9/31.3 (0.58)	31.2/31.1 (0.33)	30.6/31.1 (0.47)	30.4/31.4 (0.66)	30.2/31.1 (0.26)	31.2/31.0 (0.58)	31.7/30.9 (0.12)
Thrombocytes [N/µl]	275,622/261,543 (0.15)	238,458/270,680 (0.11)	261,658/269,553 (0.61)	278,585/264,092 (0.07)	272,953/268,234 (0.92)	265,932/269,744 (0.93)	275,569/267,233 (0.86)
GFR [ml/min]	82.3/80.9 (0.25)	85.6/81.4 (0.66)	83.9/81.4 (0.35)	82.2/81.4 (0.84)	84.6/81.3 (0.06)	82.3/81.4 (0.40)	82.5/81.4 (0.17)
Medication							
Statins	39/171 (0.13)	5/22 (0.78)	8/34 (0.56)	28/105 (0.05)	7/39 (0.89)	25/81 (***0.01***)	11/63 (0.79)
Antihypertensives	68/142 (0.55)	8/19 (1.0)	13/29 (0.99)	45/88 (0.40)	14/32 (0.92)	34/72 (0.79)	20/54 (0.41)
Anticoagulants	17/193 (0.83)	5/22 (0.05)	5/37 (0.36)	8/125 (0.34)	2/44 (0.56)	7/99 (0.59)	6/68 (0.92)
Antiplatelet drug	34/176 (0.07)	4/23 (0.77)	6/36 (0.83)	21/112 (0.28)	7/39 (0.67)	17/89 (0.32)	9/65 (0.77)
Prior Head and Neck Radiotherapy	58/152 (0.10)	12/15 (***0.01***)	12/30 (0.49)	33/100 (0.84)	15/31 (0.16)	31/75 (0.16)	18/56 (0.98)
Prior Systemic Chemotherapy	41/169 (0.30)	7/20 (0.29)	8/34 (0.80)	24/109 (0.87)	10/36 (0.43)	21/85 (0.49)	17/57 (0.18)
Prior Microvascular Free Flap	49/161 ((0.36)	8/19 (0.29)	7/81 (0.42)	29/104 (0.92)	10/36 (0.97)	30/76 (0.05)	13/61 (0.36)
Surgical experience (mean in years)	7.0/6.6 (0.13)	8.8/6.7 (***0.04***)	8.4/6.6 (***0.009***)	6.6/6.9 (0.76)	7.3/6.8 (0.28)	7.5/6.6 (0.11)	7.1/6.8 (0.62)

**Table 5:** Univariate subgroup analysis for non-osseous flaps of preoperative parameters on dependent variables. (*WHD = wound healing disorder*,* BMI = body mass index*,* ORN = osteoradionecrosis*,* MRONJ = medication associated osteonecrosis of the jaw*,* INR = International normalized Ratio*,* PTT = partial thromboplastin time*,* GFR = glomerular filtration rate*)

	Any Complication (*n* = 240) Yes/No (*p*)	Anastomosis Revision (*n* = 27) Yes/No (*p*)	Flap Loss (*n* = 17) Yes/No (*p*)	WHD (*n* = 174) Yes/No (*p*)	Partial Necrosis (*n* = 48) Yes/No (*p*)	Infection (*n* = 42) Yes/No (*p*)
Age (mean in years)	67.1/68.2 (0.27)	67.4/67.8 (0.83)	64.6/67.9 (0.44)	67.3/68.0 (0.30)	67.6/67.8 (0.84)	64.4/68.0 (***0.04***)
Female	89/151 (0.07)	11/16 (0.92)	11/6 (0.05)	65/109 (0.21)	11/37 (***0.006***)	17/25 (0.87)
Male	151/89 (0.07)	16/11 (0.92)	6/11 (0.05)	109/65 (0.21)	37/11 (***0.006***)	25/17 (0.87)
BMI (kg/m ^2^)	24.6/25.1 (0.35)	24.9/24.9 (0.60)	26.8/24.9 (0.47)	24.6/25.0 (0.46)	24.3/25.0 (0.35)	25.0/24.9 (0.70)
Comorbidities						
Nicotine abuse	85/155 (***0.01***)	6/21 (0.39)	7/10 (0.29)	58/116 (0.21)	18/30 (0.22)	10/32 (0.39)
Alcohol abuse	53/187 (***0.003***)	1/26 (0.07)	5/12 (0.15)	36/138 (0.08)	15/33 (***0.004***)	7/35 (0.98)
Hypertension	115/125 (0.90)	11/16 (0.47)	6/11 (0.30)	81/93 (0.75)	25/23 (0.52)	19/23 (0.75)
Diabetes mellitus	37/203 (0.81)	2/25 (0.41)	1/16 (0.49)	30/144 (0.32)	10/38 (0.24)	5/37 (0.56)
Arteriosclerosis	48/192 (***0.03***)	5/22 (0.79)	4/13 (0.38)	33/141 (0.22)	11/37 (0.17)	7/35 (0.83)
History of thrombosis	6/234 (0.59)	0/27 (1.0)	1/16 (0.47)	4/170 (0.79)	2/46 (0.65)	4/38 (***0.03***)
Bleeding disorder	0/240 (1.00)	0/27 (1.0)	0/17 (1.0)	0/174 (1.0)	0/48 (1.0)	0/42 (1.0)
Hypothyroidism	27/213 (0.08)	4/23 (1.0)	4/13 (0.29)	19/155 (0.16)	1/47 (***0.01***)	5/37 (0.64)
Indication						
Malignant tumor	217/23 (0.56)	25/2 (1.0)	15/2 (0.65)	161/13 (0.53)	43/5 (0.67)	33/9 (***0.007***)
ORN	8/232 (0.53)	1/26 (0.54)	1/16 (0.39)	5/169 (1.0)	2/46 (0.55)	2/40 (0.33)
Benign tumor	2/238 (0.29)	0/27 (1.0)	0/17 (1.0)	2/172 (0.18)	0/48 (1.0)	0/42 (1.0)
Osteomyelitis	2/238 (0.07)	0/27 (1.0)	0/17 (1.0)	1/173 (0.47)	0/48 (1.0)	1/42 (0.13)
MRONJ	2/238 (1.00)	0/27 (1.0)	0/17 (1.0)	1/173 (0.68)	0/48 (1.0)	1/41 (0.38)
Trauma	2/238 (0.56)	0/27 (1.0)	0/17 (1.0)	1/173 (1.0)	0/48 (1.0)	1/41 (0.18)
Other	7/233 (0.52)	1/26 (1.0)	1/16 (0.47)	3/171 (0.15)	3/45 (0.24)	4/38 (0.06)
Preoperative blood parameters						
Hemoglobin [g/dl]	13.0/13.2 (0.28)	12.4/13.2 (0.05)	12.2/13.2 (***0.01***)	13.0/13.2 (0.45)	13.2/13.1 (0.79)	12.4/13.2 (0.16)
INR	1.2/1.2 (0.50)	1.2/1.2 (0.83)	1.2/1.2 (0.75)	1.21/1.2 (0.52)	1.2/1.2 (0.95)	1.2/1.2 (0.65)
PTT [s]	31.8/31.0 (0.08)	32.0/31.3 (0.52)	30.1/31.3 (0.25)	31.7/31.2 (0.31)	32.7/31.2 (***0.04***)	32.9/31.2 (0.05)
Thrombocytes [N/µl]	266,904/286,606 (0.93)	267,625/267,994 (0.69)	277,562/267,717 (0.27)	270,161/267,179 (0.80)	252,931/269,171 (0.21)	259,722/268,507 (0.63)
GFR [ml/min]	76.9/77.4 (0.91)	80.9/77.0 (0.46)	74.1/77.3 (0.38)	76.5/77.5 (0.85)	76.6/77.4 (0.70)	76.9/77.2 (0.75)
Medication						
Statins	68/172 (0.38)	9/18 (0.40)	4/13 (1.0)	53/121 (0.16)	8/40 (0.11)	11/31 (1.0)
Antihypertensives	124/116 (0.99)	12/15 (0.45)	6/11 (0.17)	91/83 (0.86)	24/24 (0.81)	20/22 (0.59)
Anticoagulants	30/210 (0.73)	5/22 (0.39)	2/15 (1.0)	20/154 (0.51)	6/42 (1.0)	5/37 (0.81)
Antiplatelet drug	53/187 (0.85)	6/21 (0.95)	5/12 (0.39)	36/138 (0.71)	11/37 (0.83)	7/35 (0.56)
Prior Head and Neck Radiotherapy	40/200 (0.17)	6/21 (0.25)	5/12 (0.08)	24/150 (0.86)	8/40 (0.61)	11/31 (***0.02***)
Prior Systemic Chemotherapy	19/221 (0.75)	2/25 (1.0)	2/15 (0.37)	14/160 (0.74)	5/43 (0.39)	5/37 (0.23)
Prior Microvascular Free Flap	35/205 (0.25)	6/21 (0.13)	4/13 (0.17)	24/150 (0.59)	4/44 (0.35)	10/32 (***0.02***)
Surgical experience (mean in years)	5.9/6.5 (0.18)	5.3/6.3 (0.33)	6.4/6.2 (0.68)	5.6/6.5 (***0.04***)	6.5/6.2 (0.34)	6.5/6.2 (0.50)

**Table 6:** Univariate subgroup analysis for malignant surgery indications of preoperative parameters on dependent variables. (*WHD = wound healing disorder*,* BMI = body mass index*,* ORN = osteoradionecrosis*,* MRONJ = medication associated osteonecrosis of the jaw*,* INR = International normalized Ratio*,* PTT = partial thromboplastin time*,* GFR = glomerular filtration rate*)

	Any Complication (*n* = 360) Yes/No (*p*)	Anastomosis Revision (*n* = 43) Yes/No (*p*)	Flap Loss (*n* = 46) Yes/No (*p*)	WHD (*n* = 261) Yes/No (*p*)	Partial Necrosis (*n* = 75) Yes/No (*p*)	Infection (*n* = 115) Yes/No (*p*)
Age (mean in years)	65.6/67.2 (***0.02***)	64.6/66.6 (0.76)	63.4/66.7 (0.07)	66.0/66.7 (0.16)	65.4/66.6 (0.37)	63.2/67.0 (***< 0.001***)
Female	133/227 (***0.02***)	22/21 (0.19)	24/22 (0.14)	96/165 (0.06)	20/55 (***0.006***)	40/75 (0.11)
Male	227/133 (***0.02***)	21/22 (0.19)	22/24 (0.14)	165/96 (0.06)	55/20 (***0.006***)	75/40 (0.11)
BMI (kg/m ^2^)	24.2/24.9 (0.17)	23.9/24.7 (0.97)	24.9/24.6 (0.85)	24.1/24.8 (0.23)	24.0/24.7 (0.41)	23.9/24.7 (0.31)
Comorbidities						
Nicotine abuse	141/219 (***0.003***)	12/31 (0.43)	25/21 (***0.002***)	101/160 (***0.03***)	31/44 (0.13)	44/71 (0.24)
Alcohol abuse	89/271 (***< 0.001***)	3/40 (0.05)	18/28 (***< 0.001***)	67/149 (***< 0.001***)	25/50 (***< 0.001***)	29/86 (***0.04***)
Hypertension	168/192 (0.88)	20/23 (0.98)	19/27 (0.48)	122/139 (0.88)	36/39 (0.77)	52/63 (0.79)
Diabetes mellitus	55/305 (0.45)	6/37 (0.96)	8/38 (0.53)	44/217 (0.14)	13/62 (0.42)	18/97 (0.63)
Arteriosclerosis	63/297 (***0.03***)	8/35 (0.41)	9/37 (0.30)	47/214 (***0.04***)	17/58 (***0.03***)	17/98 (0.88)
History of thrombosis	12/348 (0.80)	1/42 (1.00)	3/43 (0.22)	10/251 (0.75)	3/72 (0.82)	9/106 (***0.01***)
Bleeding disorder	6/354 (0.52)	1/42 (0.45)	1/45 (0.48)	3/258 (1.00)	0/75 (0.61)	4/111 (0.06)
Hypothyroidism	54/306 (0.94)	19/33/0.13)	12/34 (***0.03***)	31/220 (0.75)	8/67 (0.31)	24/91 (0.07)
Preoperative blood parameters						
Hemoglobin [g/dl]	13.1/13.2 (0.96)	12.5/13.2 (***0.04***)	13.2/13.2 (0.81)	13.1/13.2 (0.87)	13.3/13.1 (0.57)	13.3/13.1 (0.12)
INR	1.2/1.2 (0.24)	1.2/1.2 (0.85)	1.2/1.2 (0.76)	1.2/1.2 (0.30)	1.2/1.2 (0.97)	1.2/1.2 (0.72)
PTT [s]	31.2/30.9 (0.17)	31.4/31.0 (0.24)	30.0/31.1 (0.88)	31.0/31.0 (0.55)	31.7/31.0 (0.44)	31.6/30.9 (0.08)
Thrombocytes [N/µl]	273,043/265,666 (0.17)	253,594/269,726 (0.38)	262,840/269,354 (0.94)	277,040/265,670 (0.11)	270,338/268,887 (0.79)	271,403/268,651 (0.51)
GFR [ml/min]	79.5/78.0 (0.12)	85.8/78.2 (0.07)	82.6/78.4 (0.13)	78.6/78.6 (0.48)	80.0/78.4 (0.31)	82.3/78.0 (***0.004***)
Medication						
Statins	92/268 (0.21)	11/32 (0.73)	10/36 (0.78)	73/188 (***0.04***)	12/63 (0.11)	26/89 (0.83)
Antihypertensives	169/191 (0.95)	18/25 (0.50)	16/30 (0.09)	124/137 (0.79)	36/39 (0.83)	47/68 (0.17)
Anticoagulants	37/323 (0.45)	7/36 (0.32)	5/41 (0.93)	24/237 (0.21)	5/70 (0.19)	10/105 (0.35)
Antiplatelet drug	69/291 (0.58)	8/35 (0.96)	8/38 (0.87)	47/214 (0.69)	15/60 (0.69)	18/97 (0.43)
Prior Head and Neck Radiotherapy	52/308 (0.06)	11/32 (***0.005***)	9/37 (0.10)	34/227 (0.52)	12/63 (0.26)	21/94 (***0.03***)
Prior Systemic Chemotherapy	30/330 (0.37)	4/39 (0.55)	4/42 (0.77)	23/238 (0.29)	6/69 (0.83)	14/101 (***0.04***)
Prior Microvascular Free Flap	46/314 (0.27)	9/34 (0.05)	6/40 (0.71)	35/226 (0.21)	7/68 (0.56)	21/94 (***0.01***)
Surgical experience (mean in years)	6.2/6.4 (0.88)	6.3/6.3 (0.94)	7.4/6.3 (***0.03***)	5.9/6.5 (0.20)	6.3/6.3 (0.45)	7.3/6.2 (***0.006***)

**Table 7:** Univariate subgroup analysis for non-malignant surgery indications of preoperative parameters on dependent variables. (*WHD = wound healing disorder*,* BMI = body mass index*,* ORN = osteoradionecrosis*,* MRONJ = medication associated osteonecrosis of the jaw*,* INR = International normalized Ratio*,* PTT = partial thromboplastin time*,* GFR = glomerular filtration rate*)

	Any Complication (*n* = 78) Yes/No (*p*)	Anastomosis Revision (*n* = 11) Yes/No (*p*)	Flap Loss (*n* = 13) Yes/No (*p*)	WHD (*n* = 46) Yes/No (*p*)	Partial Necrosis (*n* = 19) Yes/No (*p*)	Infection (*n* = 33) Yes/No (*p*)
Age (mean in years)	62.8/59.7 (0.60)	61.1/61.1 (0.95)	61.4/61.1 (0.97)	61.8/60.0 (0.74)	59.4/61.3 (0.20)	63.7/60.5 (0.46)
Female	34/44 (0.10)	7/4 (0.10)	8/5 (0.07)	20/26 (0.37)	11/8 (0.05)	11/22 (0.62)
Male	44/34 (0.10)	4/7 (0.10)	5/8 (0.07)	26/20 (0.37)	8/11 (0.05)	22/11 (0.62)
BMI (kg/m ^2^)	22.9/24.4 (***0.02***)	21.4/23.9 (0.05)	22.5/23.9 (0.26)	22.7/24.1 (0.06)	20.9/24.1 (***0.005***)	23.1/23.9 (0.43)
Comorbidities						
Nicotine abuse	30/48 (0.12)	0/11 (***0.02***)	3/10 (0.55)	23,723 (***0.003***)	3716 (0.10)	13/20 (0.34)
Alcohol abuse	13/65 (0.18)	0/11 (0.36)	0/13 (0.22)	11/35 (***0.009***)	3716 (0.72)	5/28 (0.77)
Hypertension	30/48 (0.34)	3/8 (0.75)	3/1 (0.55)	17/29 (0.71)	5/14 (0.42)	12/21 (0.82)
Diabetes mellitus	7/71 (0.95)	1/10 (1.00)	2/11 (0.32)	3/43 (0.76)	0/19 (0.22)	3/30 (1.00)
Arteriosclerosis	18/60 (***0.03***)	2/9 (1.00)	3/10 (0.45)	10/36 (0.26)	3/16 (1.00)	8/25 (0.20)
History of thrombosis	3/75 (0.87)	0/11 (1.00)	1/12 (0.43)	2/44 (1.00)	0/19 (1.00)	1/32 (1.00)
Bleeding disorder	2/76 (0.12)	0/11 (1.00)	0/13 (1.00)	0/46 (1.00)	0/19 (1.00)	2/31 (***0.04***)
Hypothyroidism	12/66 (0.59)	2/9 (1.00)	1/12 (0.70)	6/40 (0.40)	5/14 (0.33)	6/27 (0.85)
Preoperative blood parameters						
Hemoglobin [g/dl]	12.6/12.6 (0.81)	12.3/12.6 (0.46)	12.2/12.6 (0.31)	12.4/12.7 (0.55)	12.8/12.6 (0.99)	12.3/12.7 (0.60)
INR	1.2/1.2 (0.57)	1.3/1.2 (0.92)	1.3/1.2 (0.70)	1.2/1.2 (0.45)	1.2/1.2 (0.49)	1.2/1.2 (0.54)
PTT [s]	31.8/32.4 (0.77)	32.2/32.1 (0.92)	32.2/32.1 (0.79)	31.8/32.3 (0.87)	31.0/32.3 (0.97)	31.8/32.2 (1.00)
Thrombocytes [N/µl]	253,171/274,382 (0.30)	251,181/265,571 (0.97)	277,230/263,532 (0.51)	256,363/267,611 (0.82)	231,882/268,371 (0.15)	240,437/270,428 (0.11)
GFR [ml/min]	78.8/82.6 (0.17)	74.6/81.3 (0.22)	76.4/81.2 (0.17)	81.3/80.7 (0.99)	83.1/80.6 (0.72)	76.0/82.0 (0.10)
Medication						
Statins	14/64 (0.63)	3/8 (0.39)	2/11 (1.00)	8/38 (0.84)	3/16 (1.00)	10/23 (***0.02***)
Antihypertensives	21/57 (0.97)	2/9 (0.73)	3/10 (1.00)	12,734 (0.86)	2/17 (0.09)	7/26 (0.40)
Anticoagulants	8/70 (0.92)	3/8 (0.08)	2/11 (0.62)	4/42 (1.00)	3716 (0.41)	2/31 (0.53)
Antiplatelet drug	15/63 (0.90)	2/9 (1.00)	3/10 (0.71)	10/36 (0.55)	3/16 (1.00)	6/27 (0.92)
Prior Head and Neck Radiotherapy	43/35 (0.22)	7/4 (0.35)	8/5 (0.39)	23/23 (1.00)	11/8 (0.47)	21/12 (0.08)
Prior Systemic Chemotherapy	27/51 (0.56)	5/6/(0.34)	6/7 (0.36)	15/31 (0.97)	9/10 (0.14)	12/21 (0.58)
Prior Microvascular Free Flap	36/42 (0.17)	5/6 (0.76)	5/8 (0.87)	18/28 (0.81)	7/12 (0.72)	19/14 (***0.03***)
Surgical experience (mean in years)	7.2/7.1 (0.67)	9.9/7.0 (0.08)	9.5/7.0 (0.06)	6.8/7.3 (0.66)	9.3/6.9 (***0.03***)	6.8/7.3 (0.66)

**Table 8:** Multivariate subgroup analysis for osseous flaps on dependent variables. (*WHD = wound healing disorder*,* OR = Odds Ratio*,* CI = confidence interval*,* ROC-AUC = receiver operating characteristics – area under the curve)*

	OR [95%-CI]	*p*	ROC-AUC [95%-CI]
Any Complication			0.63 [0.58;0.69]
BMI	0.94 [0.90;0.99]	***0.02***	
Nicotine abuse	1.05 [0.65;1.68]	0.85	
Alcohol abuse	2.40 [1.30;4.44]	***0.005***	
Arteriosclerosis	1.98 [1.06;3.68]	***0.03***	
Anastomosis revision			0.73 [0.62;0.83]
Female	3.63 [1.56;8.45]	***0.003***	
Prior Head and Neck Radiotherapy	2.89 [1.25;6.56]	***0.02***	
Surgical experience	1.07 [0.99;1.16]	0.09	
Flap loss			0.68 [0.61;0.76]
Alcohol abuse	2.02 [0.98;4.14]	***0.02***	
Diabetes mellitus	2.53 [1.11;5.75]	***0.02***	
Surgical experience	1.09 [1.02;1.16]	***0.007***	
WHD			0.61 [0.57;0.64]
Nicotine abuse	1.28 [0.77;2.11]	0.34	
Alcohol abuse	2.71 [1.50;4.92]	***0.001***	
Arteriosclerosis	1.90 [1.04;3.49]	***0.04***	
Partial necrosis	-	-	-
Infection			0.67 [0.61;0.73]
Alcohol abuse	1.65 [0.96;2.84]	0.07	
Hypothyroidism	1.76 [0.99;3.12]	0.05	
Benign tumor	0.00 [0.00;0.00]	1.0	
Hemoglobin	1.13 [1.01;1.26]	***< 0.001***	
Bone exposure			0.71 [0.64;0.78]
Age	1.0 [0.97;1.02]	0.71	
Nicotine abuse	1.61 [0.85;3.05]	0.15	
Alcohol abuse	3.55 [1.79;7.02]	***< 0.001***	
Hypothyroidism	2.62 [1.39;4.95]	***0.003***	
Hemoglobin	1.16 [1.02;1.33]	***0.03***	

**Table 9:** Multivariate subgroup analysis for non-osseous flaps on dependent variables. (*WHD = wound healing disorder*,* OR = Odds Ratio*,* CI = confidence interval*,* ROC-AUC = receiver operating characteristics – area under the curve*)

	OR [95%-CI]	*p*	ROC-AUC [95%-CI]
Any complication			0.57 [0.52;0.62]
Nicotine abuse	1.22 [0.81;1.85]	0.34	
Alcohol abuse	1.59 [0.97;2.62]	0.07	
Arteriosclerosis	1.52 [0.99;2.35]	0.06	
Anastomosis revision	-	-	-
Flap loss	-	-	-
WHD	-	-	-
Partial necrosis			0.69 [0.61;0.77]
Female	0.48 [0.23;1.01]	0.05	
Alcohol abuse	2.04 [1.03;4.06]	***0.04***	
Hypothyroidism	0.16 [0.02;1.19]	0.07	
PTT	1.03 [0.98;1.08]	0.24	
Infection			0.71 [0.63;0.79]
Age	0.97 [0.95;0.99]	***0.02***	
History of Thrombosis	4.70 [1.40;15.75]	***0.02***	
Malignant tumor	0.44 [0.18;1.07]	0.07	
Prior Head and Neck Radiotherapy	1.58 [0.67;3.76]	0.30	
Prior Microvascular Free Flap	1.31 [0.53;3.24]	0.56	

**Table 10:** Multivariate subgroup analysis for malignant surgery indications on dependent variables. (*WHD = wound healing disorder*,* OR = Odds Ratio*,* CI = confidence interval*,* ROC-AUC = receiver operating characteristics – area under the curve*)

	OR [95%-CI]	*p*	ROC-AUC [95%-CI]
Any complication			0.60 [0.56;0.64]
Age	0.99 [0.98;1.00]	0.10	
Female	0.83 [0.62;1.10]	0.19	
Nicotine abuse	1.11 [0.79;1.56]	0.55	
Alcohol abuse	1.73 [1.15;2.61]	***0.008***	
Arteriosclerosis	1.56 [1.05;2.31]	***0.03***	
Anastomosis revision			0.61 [0.51;0.70]
Hemoglobin	0.86 [0.73;1.01]	0.06	
Prior Head and Neck Radiotherapy	2.19 [0.99;4.85]	0.05	
Flap Loss			0.70 [0.63;0.78]
Nicotine abuse	1.84 [0.88;3.85]	0.11	
Alcohol abuse	2.53 [1.17;5.47]	***0.02***	
Hypothyroidism	2.47 [1.21;5.04]	***0.01***	
Surgical experience	1.05 [0.99;1.10]	0.07	
WHD			0.60 [0.55;0.64]
Nicotine abuse	1.07 [0.74;1.54]	0.72	
Alcohol abuse	1.83 [1.20;2.79]	***0.005***	
Arteriosclerosis	1.29 [0.84;1.98]	0.25	
Statins	1.31 [0.92;1.88]	0.14	
Partial Necrosis			0.64 [0.57;0.70]
Female	0.57 [0.33;0.98]	***0.04***	
Alcohol abuse	2.15 [1.27;3.64]	***0.004***	
Arteriosclerosis	1.66 [0.92;2.99]	0.09	
Infection			0.66 [0.60;0.71]
Age	0.99 [0.97;1.00]	0.11	
Alcohol abuse	1.65 [1.02;2.67]	***0.04***	
History of Thrombosis	2.73 [1.18;6.32]	***0.02***	
GFR	1.01 [0.99;1.02]	0.24	
Prior Head and Neck Radiotherapy	1.25 [0.61;2.59]	0.54	
Prior Systemic Chemotherapy	1.13 [0.49;2.63]	0.77	
Prior Microvascular Free Flap	1.63 [0.90;2.96]	0.11	
Surgical experience	1.04 [1.00;1.08]	0.05	

**Table 11:** Multivariate subgroup analysis for non-malignant surgery indications on dependent variables. (*WHD = wound healing disorder*,* OR = Odds Ratio*,* CI = confidence interval*,* ROC-AUC = receiver operating characteristics – area under the curve*)

	OR [95%-CI]	*p*	ROC-AUC [95%-CI]
Any complication			0.64 [0.56;0.73]
BMI	0.92 [0.86;0.99]	***0.03***	
Arteriosclerosis	2.54 [1.08;5.97]	***0.03***	
Anastomosis revision	-	-	-
Flap Loss	-	-	-
WHD			0.63 [0.53;0.73]
Nicotine abuse	2.29 [1.00;5.20]	0.05	
Alcohol abuse	1.83 [0.63;5.32]	0.27	
Partial necrosis			0.73 [0.62;0.84]
BMI	0.85 [0.74;0.98]	***0.02***	
Surgical experience	1.11 [1.01;1.22]	***0.04***	
Infection			0.68 [0.58;0.79]
Bleeding disorder	n.a.	1.00	
Statins	2.93 [1.14;7.56]	***0.03***	
Prior Microvascular Free Flap	2.56 [1.14;5.77]	***0.02***	
